# Management of Severely Aberrant Permanent First Molars in Molar Root–Incisor Malformation Patients: Case Series and a Guideline

**DOI:** 10.3390/children8100904

**Published:** 2021-10-11

**Authors:** Ji-Soo Song, Yeon-Mi Yang, Young-Jae Kim, Jung-Wook Kim

**Affiliations:** 1 Department of Pediatric Dentistry, School of Dentistry & DRI, Seoul National University, Seoul 03080, Korea; pedosong@snu.ac.kr (J.-S.S.); neokarma@snu.ac.kr (Y.-J.K.); 2 Department of Pediatric Dentistry, School of Dentistry, Chonbuk National University, Jeonju-si 54896, Korea; pedo1997@jbnu.ac.kr; 3 Department of Molecular Genetics, School of Dentistry & DRI, Seoul National University, Seoul 03080, Korea

**Keywords:** molar root–incisor malformation, molar–incisor malformation, root malformation, cervical mineralized diaphragm, first permanent molar, extraction

## Abstract

Recently, a new type of dental anomaly, a molar–incisor malformation or molar root–incisor malformation (MRIM), was recognized. The disease phenotype is now relatively well characterized; however, its etiology and disease-mechanism need to be elucidated. The affected teeth do not respond well to conventional treatment because of severe malformation and an unusual root structure. In this study, we present the treatment of MRIM cases with the extraction of severely aberrant permanent first molars (PFMs) and suggest that the PFM extractions are performed when it is clear that third molars will develop. The purpose of this report was primarily to present amendments to the guidelines for the treatment of patients with MRIM.

## 1. Introduction

Recently, a unique form of deformity in tooth development was recognized. Its phenotypic feature was first characterized by severe dysplastic root malformations with intact crowns in all four permanent first molars (PFMs) in two affected children who had significant events (osteomyelitis or preterm birth) before the end of the first year after birth [1]. Interestingly, a plate-like structure of ectopic mineralization, named the cervical mineralized diaphragm (CMD), was identified at the level of the cementoenamel junction (CEJ); therefore, they termed it root malformation with CMD.

A subsequent report with 12 affected children extended the phenotype to the deciduous second molars and maxillary central incisors in addition to the PFMs [2]. All the affected children had systemic diseases in the brain or central nervous system at around the age of 1 to 2 years. The deciduous second molars had similar characteristics to the PFMs, but the maxillary central incisors had wedge-shaped constriction defects at the cervical region. Therefore, they called it a molar–incisor malformation to indicate defects in the PFMs and maxillary central incisors.

Later, this phenotype was further extended to other teeth, such as the permanent canines and incisors [3,4,5,6]. Moreover, the deformity was even identified in some affected individuals who did not have any remarkable past medical history in their neonatal and infant periods. Molar root–incisor malformation (MRIM) or symmetrical multiquadrant isolated dentin dysplasia were suggested as names for this newly recognized unique anomaly in tooth development [7,8].

Now, MRIM is considered a new disease entity that is characterized by localized malformation and a characteristic microstructure like CMD, and is different from other root malformations, such as hypophosphatasia and dentin dysplasia type I [1,9,10]. MRIM is very rare and its prevalence is not reported yet. To date, less than 100 cases have been reported [6]. The etiology of this rare condition is unknown at this time; however, a possible pathological link to a genetic variation was suggested [11] and a secondary effect caused by severe systemic conditions during the first 2 years of life was suggested as a local factor causing MRIM [12].

This unique deformity, regardless of its nomenclature, presents previously undescribed clinical features and it is quite difficult to get a successful conservative treatment result [13,14]. Because some experience was gained through the treatment of several cases, this report is being made for reference by other clinicians.

## 2. Cases

This study was approved by the Institutional Review Board at Seoul National University Dental Hospital (SNUDH). All five cases presented in this study were from all Korean nonconsanguineous marriages. Cases 1, 2, and 3 were included in the list of a previous report [7]. All cases were sporadic without any similar symptoms in family members.

### 2.1. Case 1

**Figure 1:**
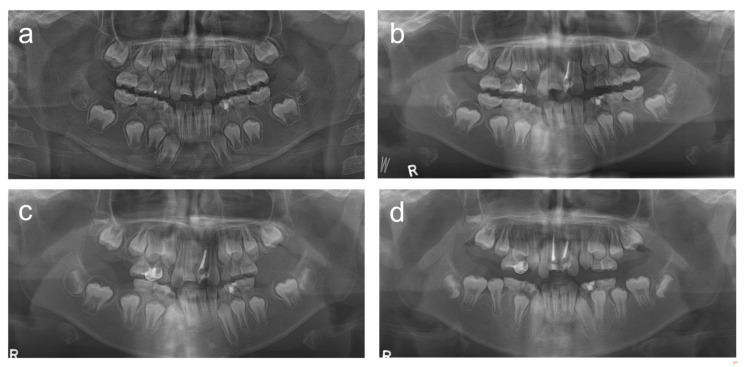

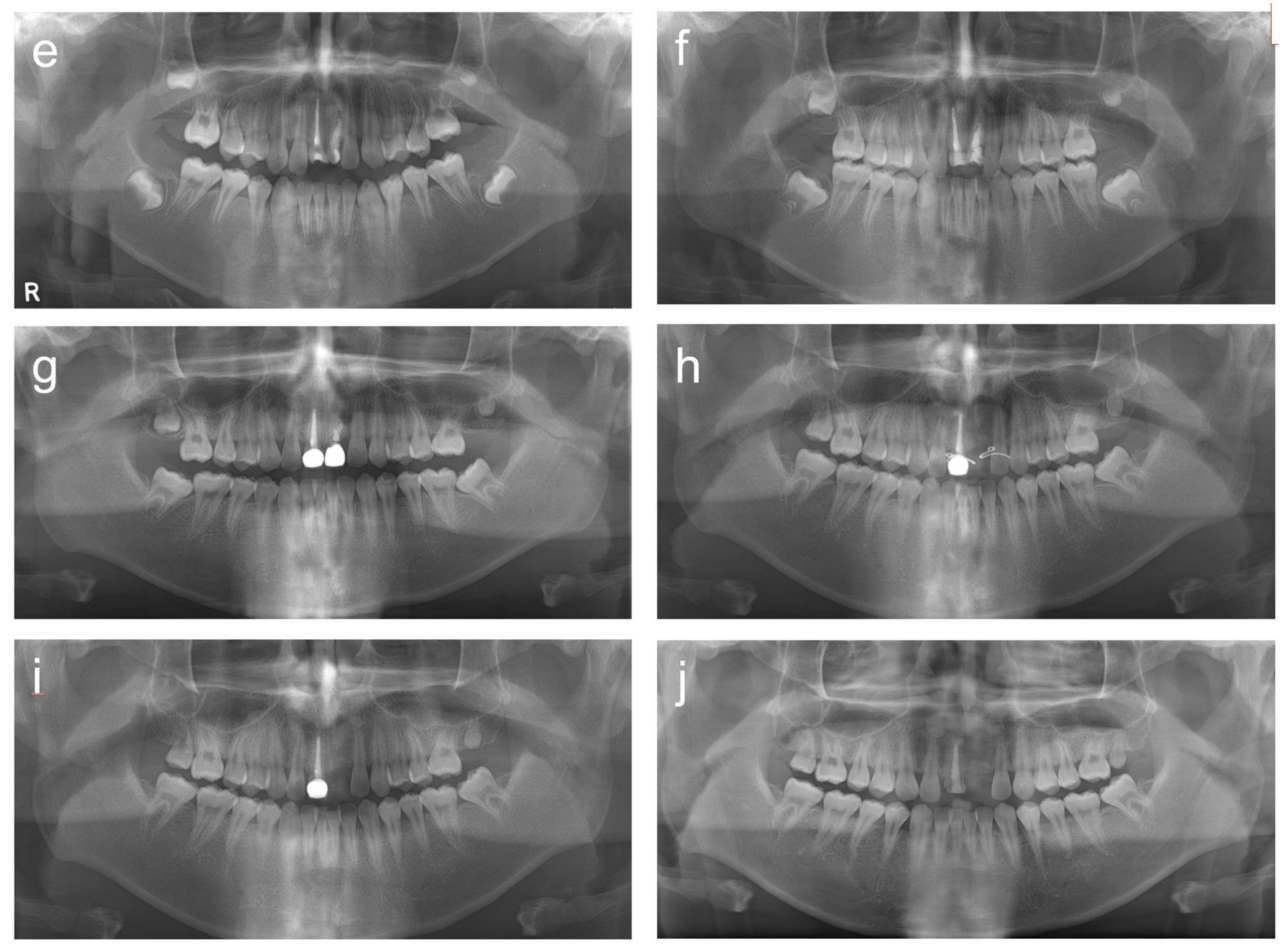
Panoramic radiographs of patient 1. (**a**) Panoramic radiograph at age 8 years 4 months. (**b**) Panoramic radiograph at age 9 years 5 months. (**c**) Panoramic radiograph at age 9 years 7 months. (**d**) Panoramic radiograph at age 10 years 5 months. (**e**) Panoramic radiograph at age 11 years 3 months. (**f**) Panoramic radiograph at age 13 years 2 months. (**g**) Panoramic radiograph at age 15 years 5 months. (**h**) Panoramic radiograph at age 16 years 7 months. (**i**) Panoramic radiograph at age 17 years 9 months. (**j**) Panoramic radiograph at age 19 years 1 month.

**Figure 2:**
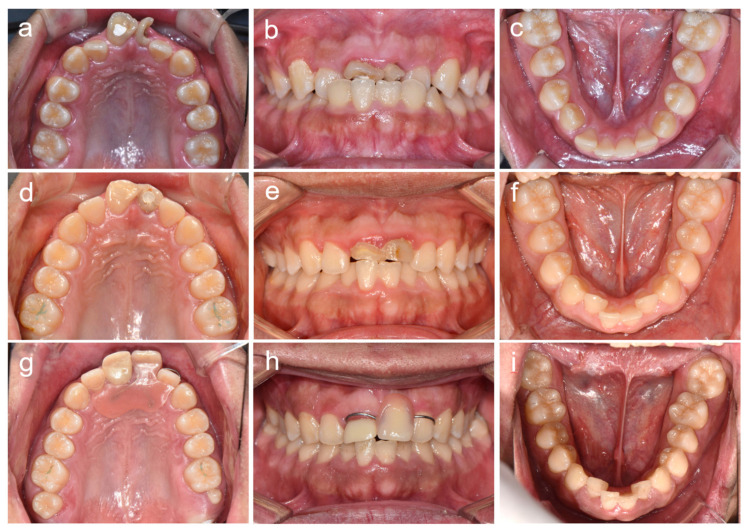
Clinical photos of patient 1. (**a**–**c**) Clinical photos at age 11 years 3 months. (**d**–**f**) Clinical photos at age 13 years 7 months. (**g**–**i**) Clinical photos at age 19 years 1 month.

The patient was an 8-year-old boy who visited the department of pediatric dentistry, SNUDH, with a complaint of an abnormal crown protrusion of the maxillary central incisors. The pregnancy and delivery by his mother were uneventful; however, he suffered from aseptic cerebral meningitis two days after birth. Two weeks later, he was hospitalized for a month due to bacterial meningitis. Clinical and radiographic examination revealed extremely short and malformed roots in all PFMs and severe constriction and dilaceration at the cervical region of the maxillary central incisors (Figure 1). Nonvital pulp treatment was performed for the maxillary central incisors; however, the left central incisor was fractured at age 15 years 5 months. Pain and discomfort were reported on the maxillary PFM at age 9 years 3 months. The development of the third molars was observed using a panoramic radiograph, even though the maxillary third molars seemed a little bit small. The right PFMs were extracted at age 9 years 7 months, and the left PFMs were extracted three weeks later. The maxillary fixed orthodontic treatment was performed to correct the anterior crossbite for 22 months from age 11 years 4 months. The maxillary third molars were small, especially the left one, but fully erupted at age 19 years 1 month (Figure 2).

### 2.2. Case 2

**Figure 3:**
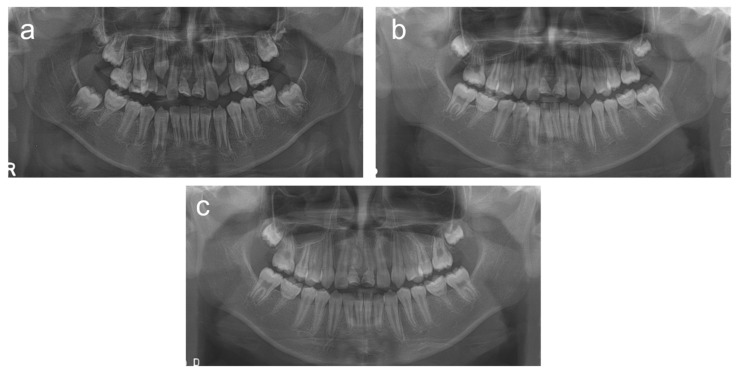
Panoramic radiographs of patient 2. (**a**) Panoramic radiograph at age 9 years 7 months. (**b**) Panoramic radiograph at age 10 years 10 months. (**c**) Panoramic radiograph at age 11 years 8 months.

The patient was a 9-year-old boy who was referred to the department of pediatric dentistry, SNUDH, for the management of root malformations in the maxillary central incisors and all PFMs. The pregnancy and delivery by his mother were uneventful, as in case 1; however, this patient also was hospitalized for 25 days due to bacterial meningitis a week after birth. During his hospitalization, magnetic resonance imaging scans were performed twice. He also had surgery for the management of otitis media at age 1 year. There were no clinical symptoms in the PFMs, but the maxillary left central incisor was fractured at the constricted part of the cervical area due to trauma four months prior to his visit. A panoramic radiograph revealed that the third molars were developing in the maxilla but not in the mandible (Figure 3). Eruption of the maxillary permanent second molars was hindered by the abnormal form of the maxillary PFMs, and the eruption space for the maxillary second premolars was not enough. Therefore, the maxillary right PFM was extracted at age 9 years 10 months, and the contralateral PFM was extracted 6 weeks later.

### 2.3. Case 3

**Figure 4:**
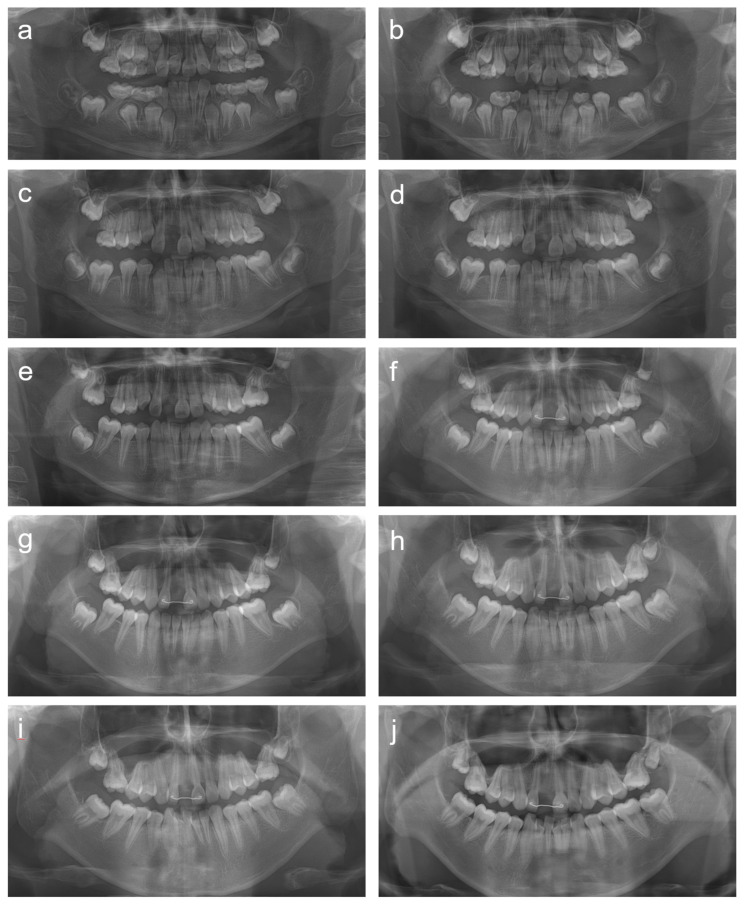
Panoramic radiographs of patient 3. (**a**) Panoramic radiograph at age 9 years. (**b**) Panoramic radiograph at age 9 years 9 months. (**c**) Panoramic radiograph at age 10 years 8 months. (**d**) Panoramic radiograph at age 11 years 2 months. (**e**) Panoramic radiograph at age 12 years 3 months. (**f**) Panoramic radiograph at age 13 years 3 months. (**g**) Panoramic radiograph at age 14 years 3 months. (**h**) Panoramic radiograph at age 15 years 3 months. (**i**) Panoramic radiograph at age 16 years 3 months. (**j**) Panoramic radiograph at age 17 years 4 months.

**Figure 5:**
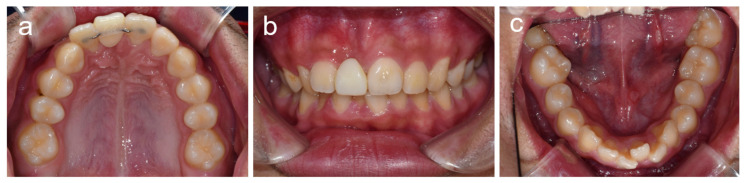
Clinical photos of patient 3. (**a**–**c**) Clinical photos at age 17 years 4 months.

The patient was a 9-year-old boy who was referred to the department of pediatric dentistry, SNUDH, for the management of root malformations in the maxillary central incisors and all the PFMs. There were no similar symptoms among the other family members, including his older brother. He was born 2 months early and hospitalized for 2 weeks due to a hydrocephalus symptom one month after birth. The development of the mandibular third molars was observed; therefore, the mandibular PFMs were extracted at age 9 years 1 or 2 months (Figure 4). The maxillary right central incisor was extracted due to infection and severe dilaceration and malformation about 7 months later. The maxillary PFMs were extracted at age 11 years 2 months after observation of the development of the maxillary third molars. The maxillary third molars were relatively small and complete eruption proceeded further at age 17 years 4 months (Figure 5).

### 2.4. Case 4

**Figure 6:**
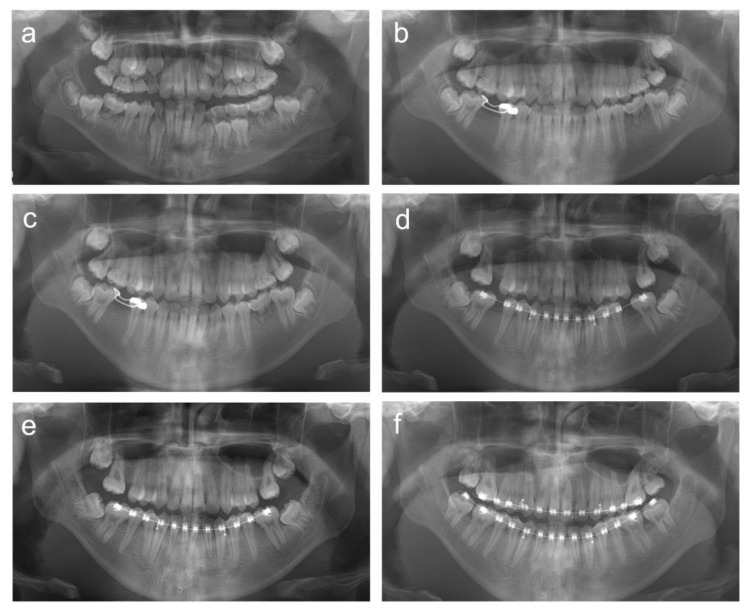
Panoramic radiographs of patient 4. (**a**) Panoramic radiograph at age 11 years 8 months. (**b**) Panoramic radiograph at age 15 years 10 months. (**c**) Panoramic radiograph at age 16 years 5 months. (**d**) Panoramic radiograph at age 17 years 5 months. (**e**) Panoramic radiograph at age 17 years 10 months. (**f**) Panoramic radiograph at age 19 years 1 month.

**Figure 7:**
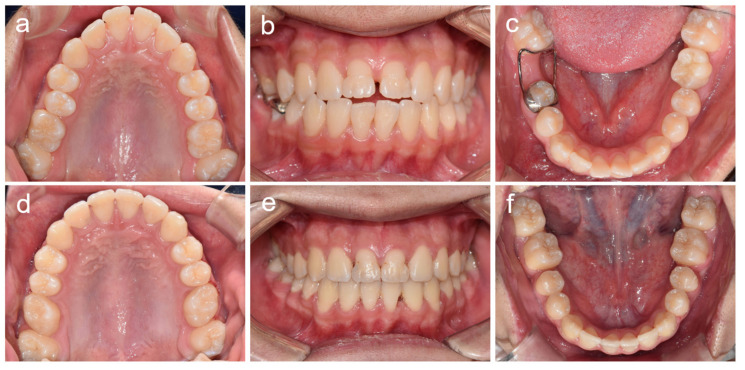
Clinical photos of patient 4. (**a**–**c**) Clinical photos at age 16 years 5 months. (**d**–**f**) Clinical photos at age 20 years 3 months.

The patient was a 15-year-old boy whose mandibular right PFM was extracted, and the space was maintained with a band and loop appliance. He was born 6 days later than the expected date of birth and hospitalized in an incubator for a month due to dyspnea and intracranial hemorrhage a few days after birth. The mandibular left PFM had a poor prognosis, and the development of third molars was observed in all four quadrants; therefore, orthodontic treatment was planned with the extraction of the remaining PFMs (Figure 6). Fixed orthodontic treatment for the mandible was started at age 16 years 6 months, and a fixed appliance for the maxilla was placed at age 18 years 3 months. Treatment was finished and the appliances were debonded at age 20 years 3 months (Figure 7).

### 2.5. Case 5

**Figure 8:**
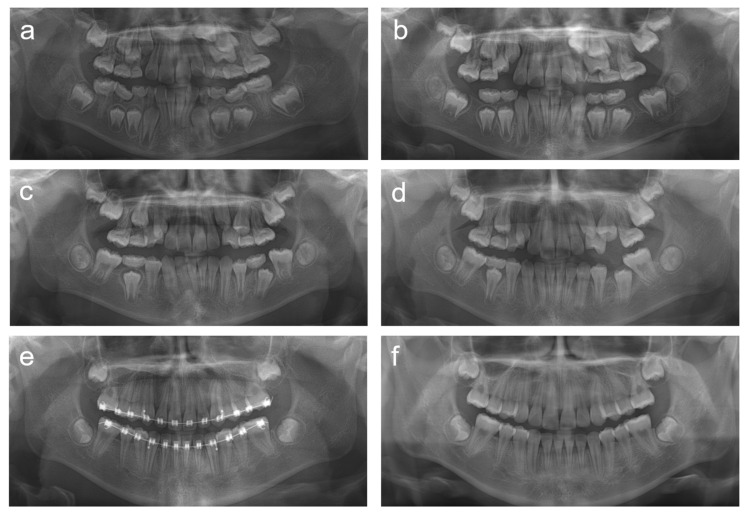
Panoramic radiographs of patient 5. (**a**) Panoramic radiograph at age 8 years 10 months. (**b**) Panoramic radiograph at age 9 years 8 months. (**c**) Panoramic radiograph at age 10 years 9 months. (**d**) Panoramic radiograph at age 11 years 2 months. (**e**) Panoramic radiograph at age 12 years 11 months. (**f**) Panoramic radiograph at age 14 years 1 month.

**Figure 9:**
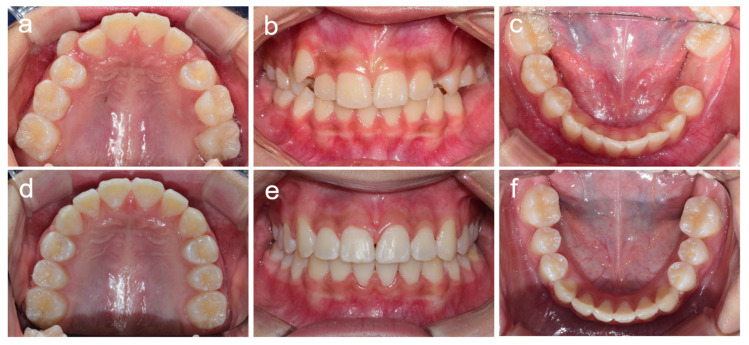
Clinical photos of patient 5. (**a**–**c**) Clinical photos at age 11 years 2 months. (**d**–**f**) Clinical photos at age 13 years 2 months.

The patient was an 8-year-old girl who was referred to the department of pediatric dentistry, SNUDH, for the management of root malformations. She was born prematurely at 27 weeks gestation and was hospitalized in an incubator for 2 months, but there were no other complications. She complained of cold sensitivity on her posterior teeth. Clinical and radiographic examination revealed root malformations in all four PFMs and deciduous second molars (Figure 8). All four third molars were observed in the panoramic radiograph; therefore, the mandibular PFMs were extracted at age 9 years 2 or 4 months. The maxillary PFMs were extracted at age 11 years 4 months, and fixed orthodontic treatments began to gain eruption spaces and to align the maxillary canines. The mandibular appliances were placed at age 11 years 11 months. Orthodontic treatment was finished at age 13 years and 2 months (Figure 9).

## 3. Discussion

Aberrant PFMs in MRIM patients can cause diverse clinical problems, such as periodontitis, spontaneous pain and abscess, complicated endodontic treatment, space loss due to early exfoliation, impaction of the PFM(s), and adjacent tooth eruption disorder [13,15]. Recommendations were suggested for the diagnosis and treatment planning of MRIM patients, and the planned and timely extraction of PFM(s) was suggested in cases of severe malformation with a poor long-term prognosis [3,16].

Of course, the PFM should be maintained and well preserved for most cases. However, the malformation frequently results in an extremely short root or unusually complicated form of root, which is very difficult to treat using conventional endodontic treatment [10,13,14]. Even with an initial anesis of inflammation or the successful control of infection, long-term survival cannot be guaranteed due to complex forms that are prone to recurrent infection [10].

However, it is worthwhile to make every effort to preserve the affected PFM before its extraction, even for short-term use with a questionable prognosis if there is no third molar development. Careful observation should be performed at regular follow-ups because the affected PFMs present complications, including periodontal bone loss, an endodontic lesion, and an endodontic-periodontal lesion. The irregular and malformed roots can be a hindrance to the eruption of the permanent second molar similar to the ectopic eruption of the PFM. Moreover, the legion can progress into the periapical cyst [15].

When the affected PFM should be extracted, the space should be maintained for the later prosthodontic or implant treatment. Space can be maintained with a fixed-type appliance, such as a band and loop; however, extrusion of an opposing tooth should be carefully prevented. Otherwise, a removable appliance with a resin tooth will provide space maintenance and occlusal support.

Identification of third molar development enables alternative treatment options. Third molars are frequently extracted due to partial or full impaction with pain and swelling or to prevent periodontal problems or damage to the permanent second molars. Surgical extraction of impacted third molars also presents complications, such as sensory nerve damage, swelling, trismus, and infection. Therefore, extraction of the affected PFMs can be positively considered to save the third molars instead of retaining the PFMs with a poor prognosis even if there are no symptoms yet.

Once the extraction of the PFMs is planned, it would be better to extract the mandibular PFM as soon as the development of third molars is recognized because it will help the bodily anterior movement of the mandibular permanent second and third molars to prevent or minimize the excessive mesial inclination that is caused by delayed PFM extraction. Periodic follow-ups using panoramic radiographs are usually sufficient in most cases for identifying and assessing the developing third molar tooth follicle.

However, the extraction of the maxillary PFMs can be delayed until the permanent second molars reach the level of the cervical area of the PFMs because the anterior movement of the maxillary permanent second molars is favorable. Extraction can be further strategically delayed beyond this point if there is no hindrance to the eruption of the maxillary permanent second molars. The extraction space can be used to align the anterior teeth if there is dental crowding.

## 4. Conclusions

In this article, we present the treatment of MRIM cases and suggest the extraction of PFMs as an alternative treatment strategy when there is the development of third molars. For the mandibular PFM, early extraction is recommended to enable better anterior bodily movement of the developing mandibular permanent second and third molars. Extraction of the maxillary PFMs can be delayed until the permanent second molars reach the level of the cervical area of the PFMs.
